# Implications of high species turnover on the south-western Australian sandplains

**DOI:** 10.1371/journal.pone.0172977

**Published:** 2017-02-28

**Authors:** Neil Gibson, Suzanne Prober, Rachel Meissner, Stephen van Leeuwen

**Affiliations:** 1 Western Australian Department of Parks and Wildlife, Science and Conservation Division, Kensington, Western Australia, Australia; 2 The University of Western Australia, Faculty of Science, School of Plant Biology, Crawley, Western Australia, Australia; 3 CSIRO, Land and Water Flagship, Wembley, Western Australia, Australia; 4 Terrestrial Ecosystem Research Network, Australian Transect Network, University of Adelaide, South Australia, Australia; Estacion Experimental de Zonas Aridas, SPAIN

## Abstract

Species turnover and its components related to replacement and nestedness form a significant element of diversity that is historically poorly accounted for in conservation planning. To inform biodiversity conservation and contribute to a broader understanding of patterns in species turnover, we undertook a floristic survey of 160 plots along an 870 km transect across oligotrophic sandplains, extending from the mesic south coast to the arid interior of south-western Australia. A nested survey design was employed to sample distances along the transect as evenly as possible. Species turnover was correlated with geographic distance at both regional and local scales, consistent with dispersal limitation being a significant driver of species turnover. When controlled for species richness, species replacement was found to be the dominant component of species turnover and was uniformly high across the transect, uncorrelated with either climatic or edaphic factors. This high replacement rate, well documented in the mega-diverse south-west, appears to also be a consistent feature of arid zone vegetation systems despite a decrease in overall species richness. Species turnover increased rapidly with increasing extent along the transect reaching an asymptote at *ca*. 50 km. These findings are consistent with earlier work in sandplain and mallee vegetation in the south-west and suggests reserve based conservation strategies are unlikely to be practicable in the south-western Australia sandplains when communities are defined by species incidence rather than dominance.

## Introduction

A detailed understanding of patterns in species turnover in species rich ecosystems has remained elusive despite their significance for conservation planning and considerable research over a number of decades. It remains unclear to what degree species turnover is driven by stochastic process and / or dispersal limitation (neutral theory) or alternatively by environmental filtering where species’ environmental tolerances are the key drivers of species turnover [1]. Past climatic regimes, long term climatic stability and evolutionary history [2–5] are also possible drivers.

Complicating matters further, the scale of individual studies has also been recognised as a contributing factor to the perceived importance of neutral vs environmental filtering to species turnover patterns [6]. Recently Barton et al. [7] suggested that species turnover shows a complex non-linear relationship between grain size (size of study plot) and geographical extent and proposed a general framework that could help explain these scale dependent results.

The extraordinarily species-rich shrublands of the Mediterranean-climate, oligotrophic sandplains of south-western Australia (kwongan) and southern Africa (fynbos) have been the focus of studies attempting to determine the relative importance of neutral processes vs environmental filtering in relation to species turnover [8–10]. These studies have focused on explaining contemporary patterns rather than accounting for the historical factors that have led to the evolution of these systems [5,11].

Fine scale soil mosaics have long been considered an important driver of species turnover in the sandplain vegetation in both regions [12–15]. However at the scale of individual soil types or vegetation units the importance of environmental filtering is much less clear, leading to suggestions these communities possess a high degree of functional redundancy which interact with recurrent fires to contribute to a lottery type dynamic within vegetation units [8,10]. Exhaustive studies of spatial point pattern of species at 0.09–0.16 ha scales in south-western Australian sandplains report significant spatial aggregation consistent with dispersal limitation within individual edaphic units [9].

To help characterise patterns of diversity, and disentangle the role of edaphic filtering from neutral processes in determining species turnover in the oligotrophic sandplains of south-western Australia, we surveyed floristic composition in edaphically uniform sites over varying geographical scales along a latitudinal / climatic gradient. At the local scale we predict that species turnover would be independent of species richness remaining constantly high across the transect in line with recent findings from other studies in the arid zone of south western Australia [16,17]. At the regional scale, we predicted species turnover would be most highly correlated with both climatic and geographic gradients, and that correlation with edaphic factors would be minimal. If dispersal limitation is a significant driver of species turnover at all scales then we predicted geographic distance (as a proximal surrogate for dispersal limitation) would also be an important explanatory variable of turnover at the local scale [18]. This same survey design allowed us to quantify the relationships between species turnover and spatial extent for a consistent spatial grain (plot size).

Specifically, we investigated four questions after controlling for edaphic variability as far as practicable. Does species turnover at the local scale remain constantly high along the latitudinal/climatic gradient despite an expected decrease in species richness? How does the pattern of species turnover change with extent along this gradient while holding spatial grain constant? Is geographic distance a significant driver of species turnover at both the regional (870 km) and local (10.5 km) scales consistent with neutral theory processes? Does the patterns of species turnover at varying scales suggest appropriate conservation strategies for south-western Australian sandplains?

## Methods

**Fig 1:**
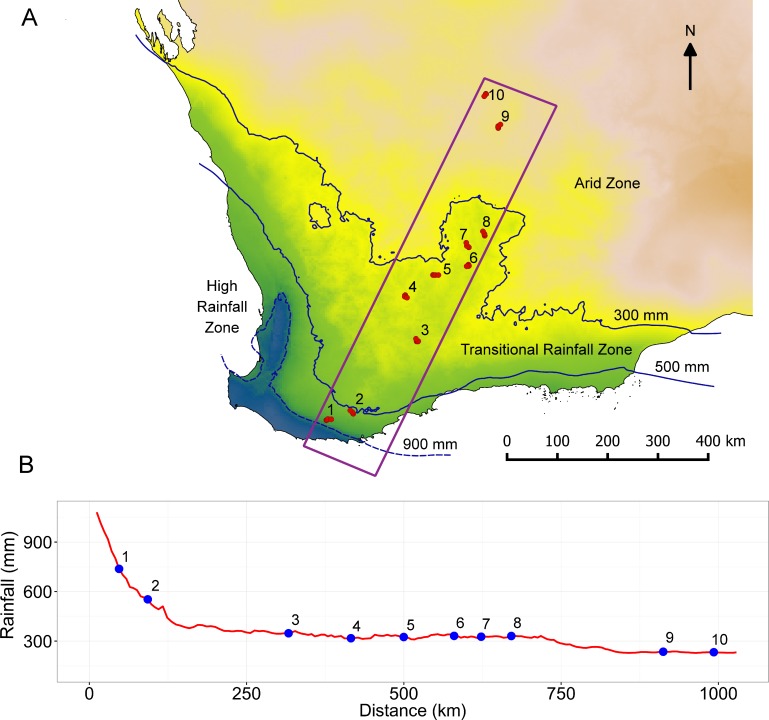
Position of transect across the sandplains in south west Australia showing the associated rainfall gradient. (A) Numbered locations indicate where sets of 16 quadrats were established. Three floristic zones were recognised based on rainfall isohyets (adapted from [19]). Background shows mean annual rainfall gradient (blue–high rainfall to brown–low rainfall), and isohyets calculated from gridded data 1961–1990 sourced from the Bureau of Meteorology [20]. (B) Profile of mean annual rainfall along vectors connecting the 10 locations (numbered circles) on the transect from south to north.

Ten sandplain locations were selected along the South West Australian Transitional Transect, a latitudinal/climatic gradient extending from the forests and woodlands of the mesic south coast to the arid sandplains of the interior (Fig 1, zones adapted from [19]). The two southern locations occur on deeply leached white sands in the High Rainfall Zone (HRZ, 500–1200 mm) supporting *Eucalyptus* Open Forest / Woodlands or Tall Proteaceous Shrubland with Mallees, the next six locations occurring on deep yellow sands in the Transitional Rainfall Zone (TRZ, 300–500 mm) largely supporting *Allocasuarina* Shrubland. The northern two locations occur on red desert sands of the Arid Zone (AZ, 200–300 mm rainfall) supporting *Eucalyptus* Open Woodland over *Triodia* Hummock Grassland.

At each location four 1 ha sites (100 x 100 m) were established at distances of *ca*. 1.5, 3 and 6 km apart. At each of the four sites four 20 x 20m quadrats were established in each corner giving a total of 16 quadrats at each location. This gave spatial scales between quadrats at each location of between 0.14–10.5 km. Distances between adjacent locations ranged from 45 to 265 km (mean 109 km, Fig 1). Sites were selected on flat or very gently sloping sandplains with a target soil depth ≥ 90 cm. Mallee vegetation and recently burnt sites (< 5 years since fire) were avoided where possible in an attempt to minimize environmental heterogeneity among samples. This sampling strategy was constrained to some extent by the availability of uncleared vegetation in the agricultural zone and access in Arid Zone.

For each quadrat, all vascular plants were recorded and collected for later identification at the Western Australian Herbarium where > 1500 voucher specimens have been lodged. Data were collected on both incidence and cover but analyses were undertaken on incidence data only. There was a high correlation between dissimilarity matrices based on incidence and cover (Spearman rank = 0.98). Range size was estimated for all species located on the transect for which more than three collections were available at the Western Australian Herbarium (> 95% species). The area of a convex hull which included all collections for each taxon was calculated using the *sp* and *geosphere* packages [21,22] in R [23]. These data were subsequently used to calculate average range size per location.

Samples of surface soil were collected from the top 10 cm of soil at 20 regularly spaced points across the quadrat. The samples were bulked and the 2 mm fraction analysed for calcium, potassium, magnesium, sodium and phosphorus using an Inductively Coupled Plasma—Atomic Emission Spectrometer (ICP-AES) as described in [24]. Electrical conductivity, organic carbon, nitrogen and pH were determined using standard methods [24]. Additional soil samples were collected at the corner of each quadrat at 90 cm depth (or immediately above any impeding gravel layer) and bulked. Average sampling depth of eight of the locations ranged from 80–90 cm while at two locations a widespread impeding layer was encountered (location 1, *ca*. 60 cm; location 3, *ca*. 30 cm). Textural analysis was undertaken on both the surface and these subsurface samples (Mean values of soil chemical and textural variables for each the 10 locations given in S1 and S2 Figs). Vegetation and soils data sets have been lodged on the AEKOS Data Portal and can be accessed via a persistent hyperlink (http://www.aekos.org.au/collection/wa.gov.au/swatt). Sites were located on lands under the control of the Department of Parks and Wildlife, Crown Land or grazing leases and the study was carried out under a Scientific Purposes Licence issued by Department of Parks and Wildlife.

### Generalised dissimilarity modelling

Generalised dissimilarity modelling (GDM) was used to fit geographic, soil and climate variables to species turnover measured by Sørensen dissimilarity measure. This technique allows turnover to be modelled as a non-linear function of environmental distance between plot pairs [25] without the need of introducing higher order polynomials to approximate the effects of geographic distance. Flexible I-splines are fitted and can then be plotted to depict the shape of the relationship between turnover and individual environmental variables. The slope of the I-splines curves indicate the rate of turnover in species composition while the height of each I-spline represents the total amount of turnover associated with the variable, holding all other variables constant. The relative importance value of individual variables can be estimated from the sum of the I-spline coefficients [26]. The linear predictor axis is referred to as predicted ecological distance and the contribution of individual variables as partial ecological distance [25,26].

Six climate variables were preselected from 17 variables extracted from the ‘one km grid’ interpolation of Hijmans [27]. They were selected based on their presumed importance in driving species turnover and were related to moisture and temperature during optimal or limiting growth periods [28]. The selected variables were mean temperature and precipitation of wettest and driest quarter, and measures of seasonality namely precipitation of warmest quarter and coefficient of variation (S3 Fig). All soil variables were included except for N which was highly correlated with organic C (r^2^ = 0.97).

Generalised dissimilarity modelling was implemented using the *gdm* package in R [29] using the default of three I-splines, with a backward elimination procedure retaining variables that made a significant contribution to explained deviance (p < 0.05) permuted 500 times at each step. The joint and individual contributions of the geographic, climate and environmental variables were calculated using variance partitioning [30]. Analyses were repeated for each of the 10 locations separately to examine the influence of scale on the importance of the explanatory variables.

### Species turnover

The related Jaccard and Sørensen dissimilarity measures have long been used to quantify differences in species composition between paired quadrats and are monotonic transformations of strict sense beta diversity (gamma/alpha) [31]. The resulting dissimilarity matrix can be averaged to provide an overall estimates of the compositional heterogeneity (beta diversity) in the data set, or be used to classify, or project the sites into reduced dimensionality. The use of the average pair-wise dissimilarity as a measure of beta diversity is somewhat suboptimal as it does not account for the variance in the pair-wise distances in the dataset [32] and gives little information on the ecological processes that have led to the differentiation between quadrats.

Baselga [33] developed multiple site dissimilarity measures (β_JAC_ and β_SOR_) based on Jaccard and Sørensen dissimilarities that are calculated across all quadrats and more importantly allow these measures to decompose into a component related to spatial turnover (β_SIM_), which is independent of species richness, and a component related to nestedness (β_NES_). Spatial turnover is caused by the replacement of species from one quadrat to another and may be a result of niche or dispersal processes. The nestedness-related component is determined by species gains or losses in nested subsets (although not a direct measure of nestedness), and can arise from such processes as selective extinction or colonisation, or habitat nestedness [31]. This decomposition of beta diversity can provide important information for conservation management as the options of how to manage a system showing high species replacement (conserve multiple examples) is opposite to strategies for managing systems with a high nestedness-related component (conserve the most species rich examples) [34].

An alternative method proposed by Podani and Schmera [35] and others decompose these dissimilarity measures into components related to replacement (albeit a different concept) and species richness differences between paired sites. These competing methodologies and conflicting terminologies have led to extensive recent literature [36–38] discussing their relative merits and differences. Applying both methodologies to our dataset showed species replacement rather than nestedness-related or species richness differences was the primary component of beta diversity; consequently Baselga’s β_SOR_ metrics was adopted.

Initially patterns in β_SOR_ were examined at scale of locations (*ca*. 10.5 km) along the transect. Subsequently the influence of extent was examined by recalculating β_SOR_ while incrementally increasing extent along the transect from south to north. For the smallest extent mean β_SOR_ was calculated for the four sites (each consisting of 4 quadrats) at location 1 (most southern location), this gave a measure of β_SOR_ at 0.141 km scale (diagonal of 1 ha site), then a single calculation of the 8 plots 1.5 km apart at location 1, and a single calculation of all 16 quadrats at location 1 gave a measure of β_SOR_ at 1.5 and 10.5 km. Subsequently the mean β_SOR_ of a 100 random samples of 16 plots was calculated first including locations 1 and 2, then 1, 2 and 3 etc till all 10 locations were included. This resampling was undertaken to ensure any effect of sample size was minimised. The geographic distance (as a measure of extent) between location 1 and the other locations was measured as the greater circle distances between the midpoints of the quadrats at each location. A repeated analysis in the reverse order from north to south gave similar results. Calculation and resampling of β_SOR_ was undertaken using *betapart* package in R [39]. The patterns shown by β_SOR_ was contrasted with that of Whittaker’s diversity measure (β_W-1_ = γ/ ᾱ - 1; where γ is gamma diversity and ᾱ is mean species richness) at the scale of individual locations and increasing extent. Whittaker’s β_W-1_ represents the effective species turnover among compositional units in the dataset in multiples of their effective species richness [40].

## Results

### Species richness patterns and range size

**Fig 2:**
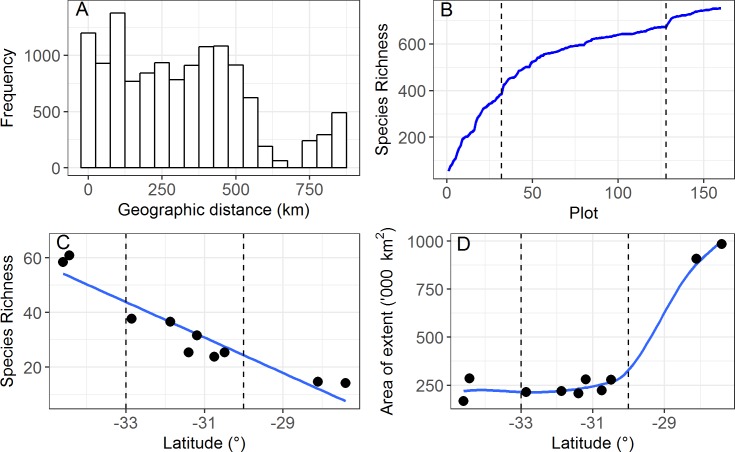
Distribution of inter-quadrat distances and patterns in species accumulation, richness, and average ranges size encountered across the transect. (A) Distribution of inter-quadrat distances. (B) Species accumulation curves from the south (high rainfall) to the north (arid). (C) Mean species richness for each of the ten locations showing trend line. (D) Average range size (km^2^) of species recorded at each of the 10 locations; loess smoothing line fitted. Vertical bars separate High Rainfall Zone (left) from Transitional Rainfall Zone (middle) and Arid Zone (right).

Reasonably uniform sampling of the geographic distances (0.14–869 km) between the 160 quadrats was achieved with the exception of distances >600 km (Fig 2A), with a total flora of 753 taxa being recorded from the 10 locations along the transect. The species pool at each location (local species pools) was estimated by the total number of species encountered in the 16 quadrats. Occurrence of singletons was highly correlated with the local species pools (adjusted-r^2^ = 0.98, p < 0.0001) with some 23% of taxa occurring a single quadrat. The number of species per quadrat is referred to as species richness but more correctly indicates species density [41].

Species accumulation from south to north was initially rapid in the HRZ (locations 1–2), became gentler in the TRZ (locations 3–8), followed by a slight increase in accumulation rate as the AZ was included (locations 9–10) (Fig 2B). Consistent with this, average species richness was highest in the south and decreased toward the north (range 60.9–14.1, Fig 2C) and was highly correlated with latitude (adjusted-r^2^ = 0.88, p < 0.0001). Local species pools decreased from 229 taxa in the south to 57 taxa in the most northern and were highly correlated with the average species richness per quadrat at locations along the transect (adjusted-r^2^ = 0.95, p < 0.0001). Average species range size (area of extent) was small (26% < 60,000 km^2^ and 50% < 140,000 km^2^) in the HRZ and TRZ but increased rapidly in the AZ (Fig 2D).

### GDM and deviance partitioning

**Fig 3:**
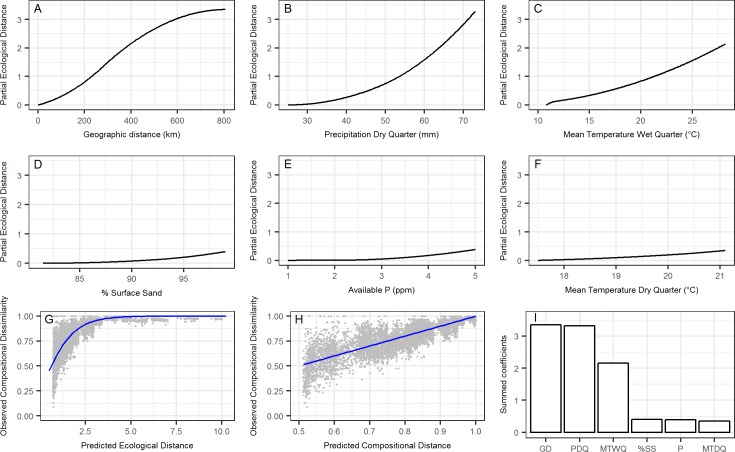
Fitted I-splines of the GDM and relative importance of predictors. Partial regression fits (A-H) of predictor variables making a significant contribution to explaining species turnover along the 870 km sandplain transect as measured by Sørensen dissimilarity. Variables arranged in decreasing order of importance value from geographic distance (A) to mean temperature of the driest quarter (F). The final three panels illustrate the relationship between: (G) observed floristic dissimilarity between each site pair in the dataset and the linear predictor of the GDM (termed ‘ecological distance’), (H) observed versus predicted dissimilarity and (I) relative importance of predictor variables determined by summing the coefficients of the I-splines from the GDM [26].

**Fig 4:**
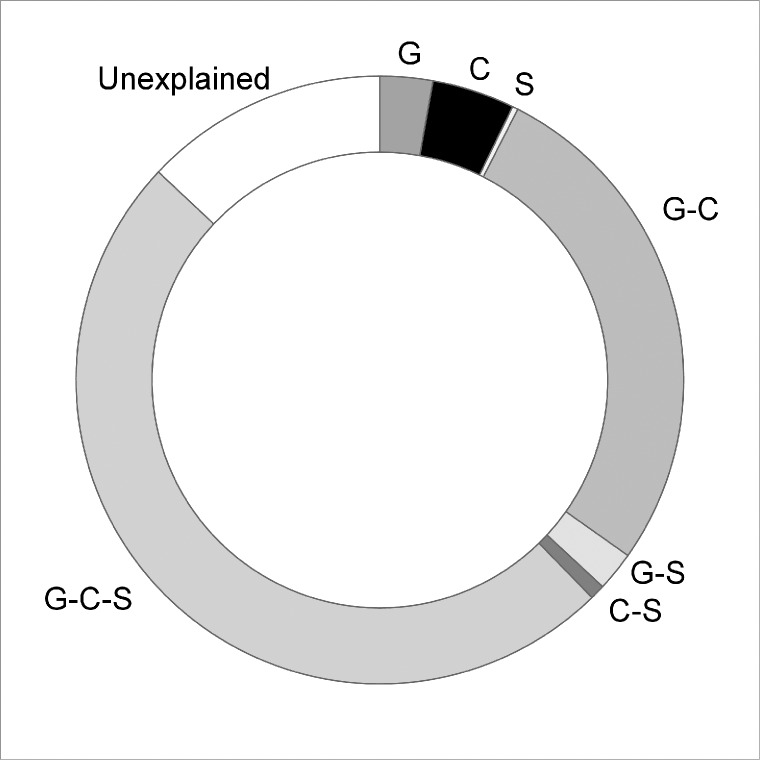
Proportion of variance of the GDM explained by the spatial, climate and soil variables across the transect. Graph showing the independent (geographic distance, G; climate, C; soil, S) and shared components (G-C, G-S, C-S, G-C-S) of the deviance explained for the fitted model across the 870 km transect. Total variance explained by the model was 87%.

The three variables that best explained species turnover as measured by Sørensen dissimilarity along the transect were geographic distance and precipitation of the driest quarter and mean temperature of the wettest quarter. Three other variables contributed to a lesser degree, percentage surface sand, available phosphorus, and mean temperature of the driest quarter (Fig 3). These six variables accounted for 87% of the null model deviance. Climate variables and geographic distance individually explained over 80% of the deviance with a joint contribution of 77.4% and independent contributions for geographic distance of 2.7% and climate of 7.6% (Fig 4). Individually soil variables explained far less of the deviance (52.7%) and had a negligible independent contribution (0.3%). Jointly geographic distance, climate and soils explained 49.6% of the deviance (Fig 4).

**Fig 5:**
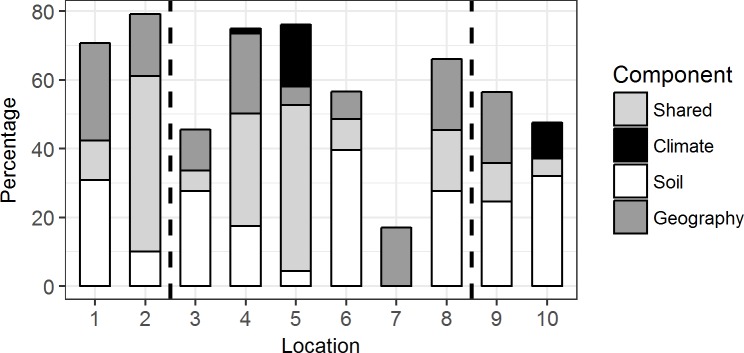
Proportion of variance of the GDM explained by the spatial, climate and soil variables at each of the 10 locations. Bar graphs showing independent (geology, dark grey; soil, white; climate, black) and shared (light grey) components of the deviance explained for the fitted models from each of the 10 locations. Models for locations 4 and 5 have significant contributions from all three variable groups and the shared components have been pooled. Vertical bars separate High Rainfall Zone (left) from Transitional Rainfall Zone (middle) and Arid Zone (right).

Somewhat different patterns were seen in the GDM models based on the 10 individual locations (Fig 5). Percentage deviance explained by the models ranged from 17–80%. Geographic distance was important in nine of the 10 models and was the sole significant explanatory variable for one model (location 7). Eight soil variables contributed significantly to nine of the ten models with between one and three soil variables being included in individual models (Table 1). While soil chemistry was expected to be a more important component in GDMs at the location scale it was somewhat surprising that soil texture (percentage sand and silt) was also an important component of six models. This may reflect subtle differences in water holding capacity that were not obvious in the field. Available P did not occur in any model (*cf*. the full model that included all 10 locations), pH was significant in two of the 10 GDMs and organic C (highly correlated with Total N) was important in three. The only climate variable, mean temperature of the wettest quarter, was also included in three of the 10 models (Table 1, Fig 5).

**Table 1:** Relative importance of predictor variables determined by summing the coefficients of the I-splines from each of the 10 location scale GDMs [26].

Predictor variables	Locations									
	High Rainfall Zone		Transitional Rainfall Zone						Arid Zone	
	1	2	3	4	5	6	7	8	9	10
Geographic distance	0.39	0.48	0.26	0.46	0.28	0.17	0.31	0.40	0.49	
% Surface sand	0.39			0.50				0.52		1.45
% Sand at depth	0.41	0.12						0.37		
% Silt at depth						0.30			0.94	
EC		0.17								
pH					0.24	0.50				
Organic C	0.27	0.29	0.53							
Mg								0.32		
Na						0.25				
Mean Temp Wettest Quarter				0.10	0.50					0.52

### Patterns in multiple site dissimilarity

**Fig 6:**
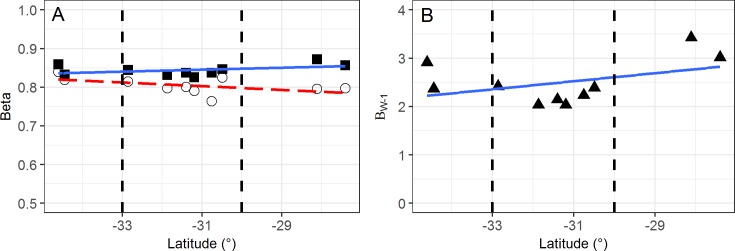
Patterns in beta diversity measures along the transect. (A) Individual location estimates (based on 16 quadrats within each location) of β_SOR_ (solid squares) were uniformly high and showed no correlation with latitude. The decreasing trend seen in the species replacement component β_SIM_ (open circles) was not significantly different from zero. (B) Patterns in Whittaker’s β_W-1_ (solid triangles) were similar, the slope was not significantly different from zero. Vertical bars separate High Rainfall Zone (left) from Transitional Rainfall Zone (middle) and Arid Zone (right).

Baselga’s multiple site dissimilarity measure (β_SOR_) was calculated for each of the 10 locations (based on 16 quadrats within each location) and decomposed into components related to species replacement (β_SIM_) and nestedness (β_NES_). At all locations β_SOR_ was uniformly high (average 0.84) as was β_SIM_ (average 0.80). Although there was slight trend for decreasing β_SIM_ with increasing latitude, the slope of the line was not significantly different from zero (F_1,8_: 3.13, p = 0.115; Fig 6A). Whittaker’s β_W-1_ showed essentially similar results with a non-significant slope and an average value of 2.5 (Fig 6B).

**Fig 7:**
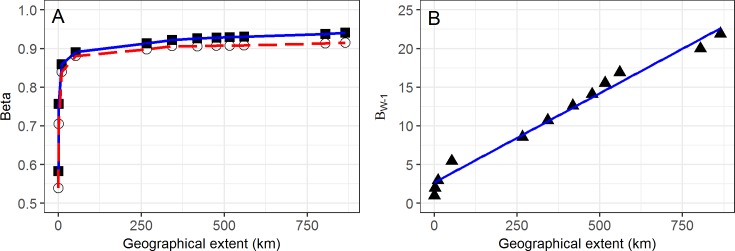
Changes in beta diversity measures with increasing linear extent. (A) Changes in β_SOR_ (solid squares) and β_SIM_ (open circles) with increasing distance to north (controlling for number of quadrats). (B) Increases in Whittaker’s β_W-1_ over the same interval.

When β_SOR_ and β_SIM_ were calculated at increasing geographical extents (within and across locations) dissimilarities increased rapidly to 10 km (β_SOR_ = 0.86), levelled off at about 50 km (β_SOR_ = 0.89) then increased only slowly until maximum extent of ca. 870 km was reached (β_SOR_ = 0.94). There was a tendency for β_SIM_ to rises at a slightly slower rate than β_SOR_ with a concomitant increase in β_NES_ with increasing geographical extent (Fig 7A). Whittaker’s β_W-1_ also increased rapidly up to ca. 10 km then in a more or less linear fashion across the rest of the transect (Fig 7B).

## Discussion

### Plant diversity across the sandplain

The wet winters and dry summers of the five main Mediterranean climate regions (south-western Australia, southern Africa, California, central Chile, and southern Europe–north Africa) have allowed the development of species rich sclerophyllous shrublands of which the kwongan for south-western Australian and the fynbos of southern Africa are regarded as most species rich [5]. Comparative studies on alpha diversity patterns have typically used standard 0.1 ha plots [42] but this size has generally been considered too onerous for wide scale floristic survey given the richness of these shrublands [43]. Comparisons in patterns in beta diversity between different regions is even more difficult where, with few exceptions [44], different sized plot, spatial extents and configurations, and numbers of different habitat types sampled vary between studies (S1 Table). The design of the current study was an initial attempt to remove the effects on environmental filtering on diversity metrics by minimizing variation in topographical and edaphic factors while sampling at a variety of scales that might provide insight into the major ecological processes operating along an extended latitudinal/climatic gradient.

The only comparable study comes from the coastal sage brush vegetation of California and Mexico (hereafter referred to as the Californian study) which used a similar grain (625 m^2^ *cf*. 400 m^2^) and extent (1000 km *cf*. 870 km, but with 40% fewer quadrats) to our transect [45,46]. The quadrats were not, however, constrained to sampling uniform topographic and edaphic sites and therefore could be expected to capture more of the diversity than those found on our transect. Further the importance of species replacement versus nestedness / species richness could not be assessed in the Californian study as access to the raw data was not available.

Obvious species richness differences were apparent between our study and the Californian study where the total flora encountered on the 1000 km Californian transect was 15% less when corrected for sampling effort and species richness was 24% lower at the quadrat scale (25 vs 32.9 taxa/quadrat). The higher richness seen in south-western Australia at both the landscape (transect) and quadrat level is considered to be primarily due to increased species accumulation resulting from more equitable climatic and stable topographical conditions during Pliocene/Pleistocene [5]. As would be expected, species turnover as measured by Whittaker’s β_W-1_ was lower along the Californian transect (14.0) compared to south-western Australia (21.9). Differences in both species richness and turnover are likely to be underestimates of true diversity differences between these regions since the Californian sites were more edaphically and topographically variable than in our study.

At the local scale (10.5 km) in our study the estimated number of complete effective species turnovers (Whittaker’s β_W-1_) was both high (averaging 2.5 from sets of 16 quadrats) and consistent at all locations along the transect. This implies a very high degree of species replacement over short distances, although it does not measure this component directly. Decomposing Baselga’s β_SOR_ allows the species replacement component (β_SIM_) to be measured directly and independently of species richness [47]. The β_SOR_ values were uniformly high at all locations (average dissimilarity of 0.84) along the transect; and species replacement was the dominant factor in changes in species composition (average β_SIM_, 0.80). The topographic and edaphic uniformity of the sites in the current study further suggests this high species replacement was not related to local scale edaphic variability or environmental filtering.

While HRZ and TRZ have long been recognised as a biodiversity hotspot [48], it has not been appreciated until recently that the high rates of species replacement seen in those regions may also be a common feature of the more species depauperate AZ. In addition to the current study, such patterns have also been documented for banded ironstone ranges in both southern and north-western Australia [16,17].

The one metric we measured that showed an abrupt change at the TRZ/AZ boundary was the mean range size which more than doubled between location 8 (TRZ) and location 9 (AZ). Gallagher’s Australia wide analysis [49] found range size gradients correlated with aridity and temperature gradients. Cook et al. [50] also suggested that the small range sizes of several pea genera in southwest Australian were related to steep environmental (rainfall and temperature) gradients. Our results are consistent with increasing range size being correlated with increasing aridity but this appears to be a step function operating close to the contemporary TRZ/AZ boundary and does not appear to be coincident with the current position of steep climatic gradients in the HRZ/TRZ. The position of these gradients would have oscillated across the south west during past glacial–interglacial cycles hence potential range size may be more related to historical area of climatically suitable habitat rather than aridity *per se*, as climatic envelopes in the south west would be necessarily small due to tighter gradients compared to broad gradients seen in central Australia [50].

The effect of increasing spatial extent while holding grain size constant led to the expected increase in β_W-1_, initially rapidly (to *ca*. 10.5 km) then in more linear fashion [7]. The rapid increase then asymptote of Baselga’s β_SOR_ (controlling for number of plots included in its calculation) appears at variance to Barton et al.’s framework [7] where β_SOR_ is expected to be high at small extents and drop as extent increases for a constant grain size. The pattern change of β_SIM_ (measuring species replacement) closely tracks β_SOR_ as would be expected from the local scale patterns. No indication of regional transitions (HRZ/TRZ/AZ) are apparent in any of these turnover/replacement metrics despite modest increases in species accumulation rates across these boundaries highlighting the high turnover rates at local and sub-regional scales.

### Environmental and geographic drivers

In the GDM, geographic distance and climatic variables account for much of the species turnover along the transect and their joint contribution was high. The most important climatic variable was precipitation in the driest quarter, in effect an aridity gradient, reinforced by contributions of mean temperature from the wettest and driest quarters. Edaphic factors, despite careful site selection, still explained a significant proportion of species turnover; however, most of this variation was spatially structured with almost no independent contribution at the regional scale. Of the edaphic factors only percentage surface sand (range 81.5–99%) and available P (range 1–5 ppm) were included in the model and their importance value was low.

Several other studies have examined species turnover in the south west using GDM or other approaches. Jones et al. [28] modelled 650 terrestrial vegetation plots of similar size to those reported here that were distributed across the TRZ while Fitzpatrick et al. [26] used herbarium collections aggregated to a 50 x 50 km grid size covering HRZ and the TRZ to examine beta diversity patterning. The model generated by Jones et al. [28], which covered a wide range of floristic and landform types, found that environmental filtering (total P, pH, Mg) and climatic gradients (precipitation of wettest and driest quarters, radiation seasonality) were the most important variables [28]. Geographic distance was included in the model but it made the lowest unique contribution of any variable. Our model examining the species turnover within a single vegetation/edaphic unit showed much less evidence of environmental filtering and a more dominant role for neutral processes (assuming geographic distance as a proximal surrogate for dispersal limitation) than that reported by Jones et al. [28].

In contrast to our results, strong environmental filtering effects (primarily pH and P) have also been reported for species richness and beta diversity in kwongan vegetation in the TRZ across a 10 km chronosequence of dune ages [51,52]. This sequence encompassed soils ages from recent to 2 million years and with a strong soil fertility gradients, not seen in our older more weathered soils [53], which showed little variation in either pH or available P. Our findings are also at variance with Sander and Wardell-Johnson study [54] which found little correlation of spatial variables (and therefore no evidence of dispersal limitation) with assemblage patterns in the forest, woodlands and swamps of the HRZ. There, environmental filtering was proposed as the main cause of species turnover. That study was undertaken over a much shorter geographical extent and, again, with significant environmental heterogeneity between quadrats.

The regional study of Fitzpatrick et al. [26] across the HRZ and TRZ showed species turnover at 50 x 50 km grain size was best explained by winter precipitation, geographical distance, available P and mean maximum temperature. This is surprisingly similar to the climatic and geographic gradient recovered in our study undertaken at a much finer spatial grain (20 x 20 m). This level aggregation appears to have largely negated confounding effects of habitat differences. An edaphic factor (available P) was included in their model but the reliability of this factor at this large grain size is questionable given the intricate soil mosaic found across south western Australia and the broad soil chemistry interpolations available.

At local scales covering (*ca*. 10.5 km) geographic distance explained considerable deviation of the models at nine of the ten locations and edaphic factors showed increased importance. Climate variables were only selected in three of the 10 models, and even in those three locations it remains questionable that climate directly affected species turnover at this scale. The observed effects possibly reflect a broad scale variable not captured in our data such as previous disturbance events like fire.

If geographic distance models dispersal limitation then its importance at both the broad and local scales is consistent with dispersal limitation being a significant factor in species turnover in these systems. While deducing process from pattern is fraught [55], the consistent identification of a factor at widely varying scales is consistent with such an interpretation [56]. In similar southern African environments little evidence was found of filtering effects along environmental gradients within vegetation classes [10], suggesting that assembly processes were largely neutral at this scale.

### Conservation implications

The two related insights of the uniformly high species replacement rate and likely dispersal limitation apparent from this analysis have significant consequence for conservation management in both kwongan (shrublands of the HRZ and TRZ) and AZ sandplain communities (dominated by hummock grasslands). If species turnover is dominated by species addition/removal (nestedness) then conservation planning can prioritize a few large areas that capture most of the species richness and significant ecological processes. If, however, species turnover is largely dominated by species replacement then the conservation strategy is both more logistically difficult and expensive as all areas are making a significant contribution to regional diversity [34]. The sandplains vegetation covered by our transect showed consistent high species turnover which was largely dominated by high species replacement. This was consistent across the HRZ, the TRZ and the AZ although there was as significant decrease in species richness with increasing aridity.

High levels of species turnover have previously been reported for studies on kwongan at the scale of single reserve (22.5 km^2^, [57]) and at a regional scales (12,000 km^2^) along the south coast, including kwongan, mallee and salt lake communities [58]. In the latter study geographic and edaphic factors were both found to be independently important in explaining species composition consistent with significant environmental filtering occurring between major edaphic units and dispersal limitation becoming dominant within individual edaphic units. To capture this variability it was proposed that a system of reserves be established for each of the broad formations at intervals of less than 15 km [58]. This has never been realised given the competing land use interests.

Our results suggest that sandplain communities are unpredictable in species composition at fine spatial scales and this scholastic element appears to result from a complex interaction between dispersal limitation (inferred from significant spatial component at all scales), an ancient stable landscape and recurrent disturbances imposed by fires as has been suggested for fynbos communities of southern Africa [8]. These processes neatly fit the view expressed by Main [59] of a biota in constant flux that he likens to a “palimpsest over-written many times with imperfect erasures”.

Therefore, conservation strategies based on acquiring representative sandplain reserves are unlikely to be achievable, at least for vegetation communities defined in terms of species incidence. The current kwongan reserves in the centres of endemism and species richness on the northern and southern sandplain are conservation jewels but are by themselves insufficient to fully represent sandplain diversity. The necessity for off-reserve management has long been recognised in regions of high beta diversity [60,61] and the long-term social values of remnant vegetation and the need of the minimising disturbance footprints, especially in light of projected climate change, are gaining traction [62].

Despite their extreme nutrient impoverishment, more than 50% of the sandplains of the HRZ and TRZ were cleared for agriculture development in the 20^th^ century [63]. In the HRZ some of the remnant sandplains have subsequently been decimated by the infection of a root rot pathogen (*Phytophorra cinnamomi*—dieback) that can eliminate a large proportion of local flora where it becomes established [64] and this epidemic remains an ongoing problem. The sandplains of the AZ have by contrast had minimal clearance and are increasingly, through Native Title processes, being returned to the ownership or management of a people who have a deep spiritual connection and cultural respect for the land [65].

Given the general lack of development pressure on both the largely intact AZ sandplains and the remnant sandplains of the south-west that have escaped the ravages of dieback, there appears to be good prospects of achieving sustainable ecological and evolutionary processes provided the general principle of consistently minimising local impacts is embraced and dieback management is continued. More challenging will be the achievement of similar long-term outcomes in formations with comparable levels of beta diversity but which occur in more productive or prospective systems [16,17].

## Supporting information

S1 Fig Variation in mean soil chemical parameters by location. Inner ring lower range limit, outer ring upper range limit (locations 1—south to 10—north).
(PDF)

S2 Fig Variation in mean soils texture by location. Inner ring lower range limit, outer ring upper range limit (locations 1—south to 10—north).
(PDF)

S3 Fig Variation in mean climate estimates by location. Inner ring lower range limit, outer ring upper range limit (locations 1—south to 10—north).
(PDF)

S1 Table Beta diversity studies from mediterranean climate regions. Comparison of extent, area, plot size, number of plots soil/vegetation unit sampled and gamma, alpha & Whittaker-1 beta diversity.
(PDF)
