# Supplementary material for: Implications of high species turnover on the south-western Australian sandplains
Source: PLoS One. 2017 Feb 28;12(2):e0172977. doi: 10.1371/journal.pone.0172977 (PMC5330496; doi:10.1371/journal.pone.0172977)
Supplement: S1 Fig — Inner ring lower range limit, outer ring upper range limit (locations 1—south to 10—north). (PDF) [file pone.0172977.s001.pdf]

Supporting Information Fig 1. Variation in mean soil chemical parameters by location. Inner ring lower range limit, outer ring upper range limit (locations 1 - south to 10 - north).

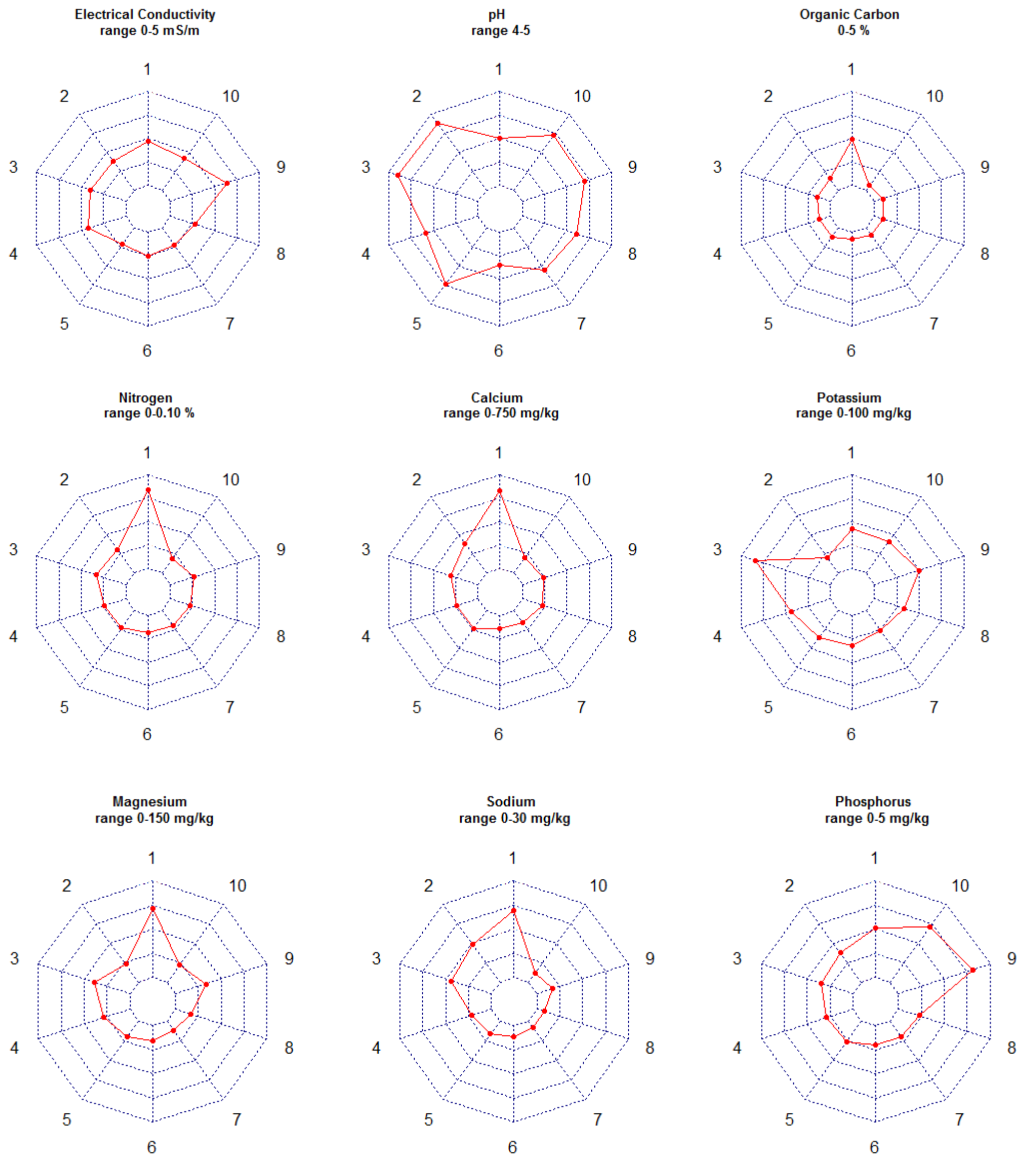

[Gibson et al. Implications of high species turnover on the south-western Australian sandplains]
