# Supplementary material for: Implications of high species turnover on the south-western Australian sandplains
Source: PLoS One. 2017 Feb 28;12(2):e0172977. doi: 10.1371/journal.pone.0172977 (PMC5330496; doi:10.1371/journal.pone.0172977)
Supplement: S1 Table — Comparison of extent, area, plot size, number of plots soil/vegetation unit sampled and gamma, alpha & Whittaker-1 beta diversity. (PDF) [file pone.0172977.s004.pdf]

Supporting Information Table 1. Beta diversity studies from mediterranean climate regions.

Comparison showing extent, area, plot size, number of plots soil/vegetation unit sampled and gamma, alpha & whittaker-1 beta diversity.
Beta diversity is strongly infulenced by grain size (plot size) and extent, and to a lesser degree by sampling effort (number of plots).

	Study area maximum extent (km)	Study area (sq km)	Plot size (sq m)	Number plots	Soil/Vegetation unit	γ	ᾱ	$\beta_{(w-1)}$		Comments / Additional data
This study									This study	
**All locations**	**850**		**400**	**160**	**Deep sand**	**753**	**32.9**	**21.9**	**All locations**	
Location 1	10.5	-	400	16	Sand (60 cm)	229	58.5	2.9	Location 1	
Location 2	10.5	-	400	16	Deep sand (90 cm)	205	60.9	2.4	Location 2	
Location 3	10.5	-	400	16	Sand (30 cm)	129	37.8	2.4	Location 3	
Location 4	10.5	-	400	16	Deep sand (90 cm)	111	36.6	2.0	Location 4	
Location 5	10.5	-	400	16	Deep sand (90 cm)	80	25.4	2.1	Location 5	
Location 6	10.5	-	400	16	Deep sand (90 cm)	96	31.6	2.0	Location 6	
Location 7	10.5	-	400	16	Deep sand (90 cm)	77	23.8	2.2	Location 7	
Location 8	10.5	-	400	16	Deep sand (90 cm)	86	25.4	2.4	Location 8	
Location 9	10.5	-	400	16	Deep sand (90 cm)	65	14.7	3.4	Location 9	
Location 10	10.5	-	400	16	Deep sand (90 cm)	57	14.2	3.0	Location 10	
Other Western Australia									Other Western Australia	
Griffin et al 1983	90	4375	100	29	Sand over laterite	317	69.8	3.5	Griffin et al 1983	
Brown 1989	350	40000	1000	20	Sand over laterite	455	91	4.0	Brown 1989	
Hnatiuk & Hopkins 1981	5	20	1000	87	3 edaphic units	338	81	3.2	Hnatiuk & Hopkins 1981	
Brown & Hopkins 1983	8	22.5	1000	13	3 edaphic units	315	80.6	2.9	Brown & Hopkins 1983	
Gibson et al. 2004	1000	230000	100:400	118	Granite	914	43.3	20.1	Gibson et al. 2004	100 sq m scored for all vascular plants, additional 300 sq m scored for overstory species.
	1000	230000	100:400	284	Duplex	1440	33.7	41.8		
	1000	230000	100:400	144	Laterite	1352	44.7	29.2		
	1000	230000	100:400	130	Deep sand	1395	43.6	31.0		
	1000	230000	100:400	139	Shrubland	1359	42.8	30.8		Same data as above by formation
	1000	230000	100:400	150	Mallee	1415	37.0	37.2		
	1000	230000	100:400	379	Woodland	1739	39.9	42.6		
										Time since fire
Gosper et al. 2012	70	3500	100	4	Mallee-heath	132	79.3	0.7	Gosper et al. 2012	2
	70	3500	100	5	Mallee-heath	148	74.8	1.0		4
	70	3500	100	6	Mallee-heath	193	82.2	1.3		6
	70	3500	100	5	Mallee-heath	146	67	1.2		19
	70	3500	100	5	Mallee-heath	125	71	0.8		25
	70	3500	100	5	Mallee-heath	119	67.2	0.8		30
	70	3500	100	5	Mallee-heath	127	65.2	0.9		35
	70	3500	100	5	Mallee-heath	134	66.2	1.0		45
	70	3500	100	8	Mallee-heath	154	64.1	1.4		55+
	70	3500	100	5	Mallee	121	41.4	1.9		4
	70	3500	100	5	Mallee	130	42.4	2.1		6
	70	3500	100	5	Mallee	100	38.8	1.6		19
	70	3500	100	5	Mallee	88	40.2	1.2		25
	70	3500	100	5	Mallee	95	38.6	1.5		30
	70	3500	100	5	Mallee	89	34.6	1.6		35
	70	3500	100	5	Mallee	80	30.2	1.6		45
	70	3500	100	5	Mallee	108	37.2	1.9		55+
South Africa SW Cape									South Africa SW Cape	
Cowling 1990									Cowling 1990	
Geelrug transect	< 5	-	1000	5	3 vegetation units/ 5 edaphic units	369	91	3.1	Geelrug transect	
Hagelkraal transect	< 5	-	1000	5	3 vegetation units/ 5 edaphic units	249	55.6	3.5	Hagelkraal transect	
Soetanysberg transect	< 5	-	1000	5	5 vegetation units/ 4 edaphic units	235	58.2	3.0	Soetanysberg transect	
Thuiller et al. 2007	60	77.5	50	81	7 vegetation units	428	38.4	10.1	Thuiller et al. 2007	
	60	77.5	50	34	Mesic oligotrophic proteoid fynbos A	257	48.7	4.3		
South Africa SE Cape									South Africa SE Cape	
Cowling 1983 SE Cape		1700			Cape Fynbos Shrublands				Cowling 1983 SE Cape	
					SE Mountain Fynbos					
	<50	-	100	8	Tetraria-Thamnus	87	26.4	2.3		

	<50	-	100	5	Leucospermum-Tetraria	56	26.6	1.1		
					Grassy fynbos					
	<50	-	100	5	Thamnochortus-Eriea	69	33.8	1.0		
	<50	-	100	9	Protea-Clutia	128	43.5	1.9		
	<50	-	100	16	Erica-Traehypogon	212	40.1	4.3		
	<50	-	100	4	Themeda-Passerina	92	36.3	1.5		
	<50	-	100	7	Thamnoehortus-Tristachya	133	46.6	1.9		
					South Coast Dune Fynbos					
	<50	-	100	13	Restio-Agathosma	144	32.4	3.4		
	<50	-	100	12	Restio-Maytenus	114	33.5	2.4		
	<50	-	100	5	Themeda-Stenotaphrum	104	40.2	1.6		
					Cape Transitional Small-leaved Shrublands					
					South Coast Renosterveld					
	<50	-	100	11	Themeda-Cliffortia	120	33.6	2.6		
	<50	-	100	10	Elytropappus-Eustaehys	162	51.5	2.1		
	<50	-	100	12	Elytropappus-Metalasia	152	40.8	2.7		
	<50	-	100	4	Elytropappus-Relhania	97	42.5	1.3		
					Afromontane Forest					
					Knysna Afromontane Forest					
	<50	-	100	5	Rapanea-Canthium	71	32.8	1.2		
	<50	-	100	4	Rapanea-Ocotea	34	21.2	0.6		
					Subtropical Transitional Thicket					
					Kaffrarian Thicket					
	<50	-	100	11	Pterocelastrus-Gonioma	107	36.9	1.9		
	<50	-	100	18	Pterocelastrus-Euclea	130	37.3	2.5		
	<50	-	100	12	Cassine-Cussonia	93	24.6	2.8		
	<50	-	100	5	Cassine-Schotia	67	29.6	1.3		
					Kaffrarian Succulent Thicket					
	<50	-	100	15	Sideroxylon-Euphorbia	120	35.8	2.4		
	<50	-	100	5	Euclea-Brachylaena	108	45	1.4		
California									California	
**Westman 1981**	**1000**	-	**625**	**67**	**Californian Coastal Sage Scrub**	**375**	**25**	**14.0**	**Westman 1981**	
Westman 1983	400	-	625	13	Diablan	173	30	4.8	Westman 1983	
	330	-	625	21	Venturan I	127	18	6.1		
	330	-	625	13	Venturan II	105	20	4.3		
	330	-	625	19	Riversidian	198	29	5.8		
	330	-	625	12	Diegan	138	26	4.3		
	200	-	625	8	Martirian	153	41	2.7		
	100	-	625	7	Vizcainan	129	33	2.9		
Whittaker et al 1979	< 70	-	1000	10	Open oak woodland	106	29.6	2.6	Whittaker et al 1979	Santa Catalina Mts
	< 70	-	1000	10	Pygmy conifer-oak scrub	89	24.1	2.7		Santa Catalina Mts
Israel									Israel	
Finkel et al 2001 Israel	<0.5	-	1000	3	garigue / dry open park forest	205	118	0.7	Finkel et al 2001 Israel	EC II - south facing slope
	<0.5	-	1000	3	dense forest	54	43.7	0.2		EC II - north facing slope
	<0.5	-	1000	3	open park forest / savanna grassland	175	125.3	0.4		ECI - south facing slope
	<0.5	-	1000	3	dense maqui forest	191	112.3	0.7		ECI - north facing slope
France									France	
Capitanio & Carcaillet 2008	< 1 for individual locations	218	100	20/location	Aleppo pine forest	40-75	10-15	3-5	Capitanio & Carcaillet 2008	6 locations with different time since fire, figures estimated from graphs
Tuscany									Tuscany	
Chiarucci et al 2001	0.05	0.25	100	10	Q. ilex forest	27	11.6	1.3	Chiarucci et al 2001	
	0.05	0.25	100	10	Q. ilex forest	39	18.2	1.1		
	0.05	0.25	100	10	Q. cerris forest	86	43.3	1.0		
	0.05	0.25	100	10	Q. cerris forest	88	49.4	0.8		
	0.05	0.25	100	10	F. sylvatica forest	22	7.8	1.8		
	0.05	0.25	100	10	F. sylvatica forest	31	12.2	1.5		

References

Brown JM (1989) Aust J Ecol 14:345–355. doi: 10.1111/j.1442-9993.1989.tb01443.x
Brown JM, Hopkins AJM (1983) Aust J Ecol 8:63–73. doi: 10.1111/j.1442-9993.1983.tb01519.x
Capitanio R, Carcaillet C (2008) For Ecol Manag 255:431–439. doi: 10.1016/j.foreco.2007.09.010
Chiarucci A, Enright NJ, Perry GLW, et al (2003) Divers Distrib 9:283–295.
Cowling RM (1983) Vegetatio 54:103–127. doi: 10.1007/BF00035145
Cowling RM (1990) J Veg Sci 1:699–710. doi: 10.2307/3235578
Finkel M, Fragman O, Nevo E (2001) Isr J Plant Sci 49:285–295.
Gibson N, Keighery GJ, Lyons MN, Webb A (2004) Rec West Aust Mus Suppl 67:139–189.
Gosper CR, Yates CJ, Prober SM, Parsons BC (2012) Austral Ecol 37:164–174. doi: 10.1111/j.1442-9993.2011.02259.x
Griffin EA, Hopkins AJM, Hnatiuk RJ (1983) Vegetatio 52:103–127.
Hnatiuk R, Hopkins A (1981) Aust J Ecol 6:423–438.
Thuiller W, Slingsby JA, Privett SDJ, Cowling RM (2007) PLoS ONE 2:e938. doi: 10.1371/journal.pone.0000938
Westman WE (1983) Vegetatio 52:3–19.
Westman WE (1981) Ecology 62:170–184. doi: 10.2307/1936680
Whittaker RH, Niering WA, Crisp MD (1979) Vegetatio 39:65–76. doi: 10.1007/BF00052018

[From: Gibson et al. Implications of high species turnover on the south-western Australian sandplains]
